# Prevalence of tuberculosis in Ethiopia: an umbrella review of systematic review and meta-analysis

**DOI:** 10.3389/fpubh.2025.1555901

**Published:** 2025-11-05

**Authors:** Wagaw Abebe, Solomon Gedifie, Tadele Emagneneh

**Affiliations:** ^1^Department of Medical Laboratory Science, College of Health Sciences, Woldia University, Woldia, Ethiopia; ^2^Department of Midwifery, College of Health Sciences, Woldia University, Woldia, Ethiopia

**Keywords:** prevalence, tuberculosis, umbrella review, Ethiopia, TB

## Abstract

**Background:**

Tuberculosis is one of infectious disease, which caused by *mycobacterium tuberculosis*. It is still one of the major problems threatening public health worldwide. However, there are inadequate systematic studies and statistical assessments of tuberculosis prevalence, mainly in Ethiopia.

**Objective:**

This umbrella review aimed to determine the prevalence of tuberculosis across the country.

**Methods:**

In accordance with standard review principles, a systematic search was conducted on Web of Science, PubMed, Science Direct, and Google Scholar to find pertinent studies. For the final umbrella review, a total of eleven relevant studies on tuberculosis prevalence were found. Data was extracted using Microsoft Excel with separate sheets for tuberculosis. The extracted data was analyzed with STATA software version 17.0. A sensitivity analysis was carried out to assess the role of each study in the final results. The Egger’s test and a funnel plot were used to assess the existence of publication bias. Trim and fill analysis were used for determining a bias-adjusted effect estimate. Inverse of variance statistics were used to assess heterogeneity among studies. If the I^2^ value was ≥50%, significant heterogeneity was identified, and subgroup analysis was carried out.

**Results:**

This umbrella review includes 11 studies. In this review, the pooled prevalence of tuberculosis was 12.81% (95% confidence interval, 5.07–20.55). The subgroup analysis based on sample size and number of studies revealed significant variations in the pooled tuberculosis prevalence.

**Conclusion:**

This comprehensive study revealed that tuberculosis is widespread in Ethiopia. The observed increase in tuberculosis incidence emphasizes the important need for improved infection prevention and monitoring systems to reduce tuberculosis burden and transmission in Ethiopia. Furthermore, collaboration at the local, national, and international levels is required to address the factors that lead to tuberculosis and mitigate its impact on public health. Additionally, it provides crucial information to Ethiopian government officials and administrators.

## Introduction

Tuberculosis (TB) is caused by the *Mycobacterium tuberculosis* and is still one of the major problems threatening public health worldwide (1). It is a transmissible disease that is a major cause of ill health, one of the top ten causes of mortality worldwide, and the largest cause of death from a single infectious agent, even ranking higher than HIV/AIDS (2). Globally, 8.2 million people were newly diagnosed with TB in 2023, an increase from 7.5 million in 2022 and 7.1 million in 2019, and much beyond the figures of 5,800,000 in 2020 and 6,400,000 in 2021. In 2022 and 2023, there was likely a significant backlog of patients who got tuberculosis but were not detected or treated due to COVID related interruptions (3).

According to the World Health Organization’s global TB report, 10.6 million people will be affected with TB by 2021, with Africa accounting for 2.5 million of those cases. In the same year, 1.7 million people died from TB worldwide, including 417,000 deaths (more than 25%) in Africa (4). The highest TB burden is reported in Sub-Saharan Africa (SSA), where the estimated incidence rate is 201 per 100,000 individuals. Furthermore, the African continent has a 3.6% prevalence rate of drug resistant TB (5).

World Health Organization has launched control measures, including directly observed treatment short courses (DOTS), as an approach (6). Following its introduction, DOTS was endorsed by international TB authorities. DOTS has proven to be effective in obtaining a high level of treatment success and has become an essential indicator for assessing the effectiveness of TB control efforts (7, 8). Early detection and appropriate treatment of TB are key components of the global DOTS strategy. The targets for controlling the global epidemic, as acknowledged by the WHO/Stop TB Partnership and included in the Millennium Development Goals (MDGs), to diagnose at least 70% of infectious cases and successfully treat at least 85% (6).

The first Ethiopian national population based TB prevalence survey indicates that over half a century ago, TB was identified as a significant public health issue in Ethiopia (9). TB, exacerbated by the HIV/AIDS epidemic, remains a severe public health issue in Ethiopia (10). According to Ethiopia’s national population-based survey from 2010/11, the prevalence of all kinds of TB was 224 per 100,000 people (9). With this prevalence, Ethiopia ranks seventh out of 22 countries with a high TB burden. In the early 1990s, Ethiopia adopted DOTS as a TB preventive and control strategy in response to this burden (4). DOTS has been estimated to have 100% geographical coverage, however it is only found in 95% of health facilities (10, 11).

Risk factors for these TB related outcomes include behaviors, structures, and other illnesses that are frequently linked to poverty, such as HIV, poor nutrition, smoking, alcoholism, diabetes, crowded living or working situations, and exposure to indoor air pollution (12, 13). The ambitious goal of the Sustainable Development Goal (SDG) is to eradicate the TB epidemic by 2030. To this purpose, quantifiable benchmarks have been established, such as a 10% yearly decrease in the prevalence of TB worldwide by 2025. In order to support policy and program decision making, it is imperative that progress toward this SDG target be thoroughly assessed, tracked, and evaluated (14).

Previous studies have various limitations that impact the generalizability of their findings. These consisted mostly of cross-sectional studies, which presented snapshots of the situation rather than demonstrating the dynamic character of the study population. The aggregated results might be influenced by the various diagnostic techniques for TB used in these studies (15–23). To overcome these shortcomings, this umbrella review develops on previous reviews by investigating the studies and data sources. The future studies should overcome the gaps found in previous reviews by covering both published and unpublished research as well as studies in different languages. Furthermore, this study takes a more comprehensive approach to assess the TB burden in Ethiopia, using a more robust methodology. This strengthens the findings and offers a more complete picture of the TB situation in Ethiopia. Overall, while past shortcomings are acknowledged, this review provides a better picture of the incidence of TB in the country.

Furthermore, there is insufficient comprehensive systematic study and statistical assessments of TB prevalence, particularly in Ethiopia. As a result, the aim of this umbrella review was to compile the heterogeneous findings of systematic reviews and meta-analysis (SRMA) studies on the burden of TB into a single comprehensive document that allows the results of these reviews to be compared and contrasted. To the best of our knowledge, no umbrella review has been conducted to determine the weighted pooled prevalence of TB in Ethiopia. Thus, data from this review will offer insights that help health professionals and policymakers to design evidence-based preventive and control strategies for TB. Therefore, the purpose of this umbrella review was to determine the pooled prevalence of TB in Ethiopia.

## Method

### Protocol, registration, and study design

This umbrella review’s protocol was already designed and registered (PROSPERO: CRD42025631162). We conducted a comprehensive analysis of research describing the prevalence of TB using umbrella reviews.

### Database and search strategy

Initial searches for this umbrella review began on November 1, 2024. This umbrella review included studies completed in Ethiopia up to December 30, 2024, which were published in English. To find research regarding the prevalence of TB reported among the Ethiopian population of different study subjects, an inclusive literature search was carried out. Both electronic and gray literatures were systematically searched. Data was retrieved using PubMed, Science Direct, Web of Science, and Google Scholar. In addition to search terms alone, Boolean operators such as “OR” and “AND” have been utilized. Google Scholar utilized the following keywords, for instance, to find pertinent studies: [“Prevalence” OR “epidemiology” AND “tuberculosis” OR “TB” OR “*Mycobacterium tuberculosis*” OR “*M. tuberculosis*” OR “MTB”AND “systematic review” OR “meta-analysis” OR “synthesis” AND “Ethiopia” 2014–2024]. The citation lists of the included studies were also subjected to a snowball search. Studies that were recorded between January 1, 2014 and December 30, 2024, were included by the researcher.

### Eligibility criteria

The EndNote version 20 reference management program (Tomson Reuters, New York, NY) was used to import articles from the aforementioned databases. The included studies for this comprehensive evaluation were: (1) Meta-analyses and systematic review studies that provided a TB prevalence (2) Articles published on Peer-reviewed journal; and (3) Articles published by English-language from January 1, 2014, to December 30, 2024. Studies were excluded if: (1) Articles scored poorly on the aforementioned quality criteria. (2) Letters, observations, editorials, and case series: and/or failed to assess the intended outcome (i.e., the prevalence of TB); (3) Studies which are written other than systematic reviews and meta-analyses guideline were excluded; (4) Studies that did not offer enough data for pooling prevalence of TB were excluded.

### Outcome of interest

The prevalence of TB in Ethiopia was the primary outcomes of interest. These were given in the systematic reviews and meta-analyses as a percentage and as the number of cases (n)/total number of participants (N).

### Study selection and quality assessment

The assessing the methodological quality of systematic reviews (AMSTAR) tool was used to evaluate the studies’ quality (24). The titles retrieved in the aforementioned databases have been evaluated by two independent reviewers (WA and SG). Following that, two reviewers (WA and TE) independently evaluated relevant studies for abstracts. Finally, three reviewers (WA, TE, and SG) screened the complete text. The nine critical appraisal checklists were used to evaluate the quality of systematic review papers. This review comprises papers with final quality scores of at least 50%.

### Data extraction

Relevant data was gathered or recorded from each possible study using a standardized data extraction form in Microsoft Excel 2010. The extraction procedure collected data on a variety of categories, including research parameters such as first author, year of publication, study population, study design, number of participants, study area/region, tuberculosis prevalence, and associated factors. Two reviewers (WA and TE) evaluated the extracted data for accuracy and consistency. The third reviewer (SG) was also consulted as necessary.

### Statistical analysis

Stata version 17 (Stata Corp. Stata Statistical Software; College Station, TX: Stata Corp LP) was used to examine the retrieved data after it was imported into Microsoft Excel. A random-effects model was used to generate a summary estimate of the prevalence across studies. The point estimate was used with a 95% confidence interval. Sensitivity analysis was performed to analyze the role of each study in the final conclusion by omitting each study one at a time, and the presence of publication bias was determined by visually evaluating funnel plots and using Egger’s test. A bias-adjusted effect estimate was obtained by performing trim and fill analysis technique. Inverse of variance (I^2^) statistics were used to assess study heterogeneity (25). A value of ≥50% was considered significant. To identify sources of heterogeneity in studies with significant differences (I^2^ ≥ 5 0%), we conducted subgroup analyses.

## Result

### Searching results

**Figure 1:**
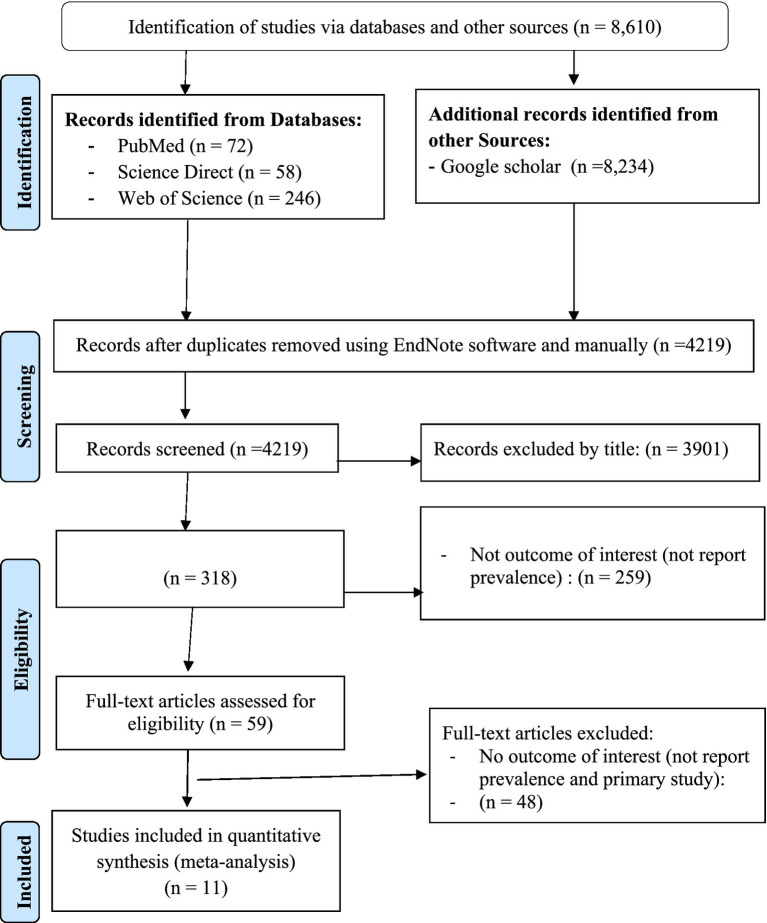
PRISMA flow diagram indicated the results of the search and reasons for exclusion (63).

In this umbrella review, a total of 8,610 studies were retrieved from searched electronic databases and other sources, such as Google Scholar. Of the total, 4,219 articles were non-duplicated and subjected to further evaluation. The 4,171 articles were assessed and excluded after reviewing their title, abstract, and other reasons (duplicate studies, primary studies, and others), while 59 articles were retained for full-text evaluation. After a full-text review, the final umbrella review included 11 potential articles (15–23, 26, 27) that reported on the prevalence of TB in Ethiopia (Figure 1).

### Descriptive summary of included studies

#### Characteristics of the included studies

Most systematic review and meta-analysis studies included in this review were cross sectional studies. Also, they included a total of 208 studies, providing a total sample of 701,148 study participants. The number of studies per systematic review and meta-analysis ranged from 9 (lowest) (17) to 53 (highest) (18). The sample size per meta-analysis ranged 4,044 (lowest) (26) to 354,613 (highest) (27). The overall characteristics of the included studies, including the topic they addressed, are shown in (Table 1).

**Table 1:** Characteristics of the included studies in an umbrella review.

Author	Year of publication	Country	Sample size	Number of article	Study design	TB prevalence (%)	Quality
Amere et al. (23)	2024	Ethiopia	24,020	21	Cross sectional and random trial study design	9.84	14
Melese et al. (19)	2024	Ethiopia	32,909	34	Cross sectional study design	11.7	13
Fassikaw et al. (21)	2024	Ethiopia	6,668	13	Cohort and retrospective study design	12.1	14
Kefyalew et al. (27)	2023	Ethiopia	354,613	13	Cross sectional study design	0.19	15
Ayinalem et al. (18)	2023	Ethiopia	12,772	53	Cross sectional, retrospective cohort, and Prospective study design	30.2	14
Getu et al. (20)	2022	Ethiopia	5,439	20	Cross sectional and retrospective study design	43	13
Ayinalem et al. (26)	2021	Ethiopia	4,044	14	Cross sectional and retrospective cohort design	4.14	14
Demeke et al. (15)	2020	Ethiopia	10,074	10	Cohort study design	16.58	15
Balew et al. (17)	2019	Ethiopia	237,648	9	Cross sectional design	0.11	15
Melkalem et al. (22)	2019	Ethiopia	8,875	11	Retrospective study	4.8	14
Addisu et al. (16)	2017	Ethiopia	4,086	10	Cross sectional study design	8.33	14

#### Heterogeneity of included studies

The prevalence of TB was estimated using heterogeneity analysis. There was substantial variability in the prevalence of TB, with I^2^ statistics indicating more than or equal to 100% at *p* = 0.00 (15–23, 26, 27).

#### Publication bias of included studies

**Figure 2:**
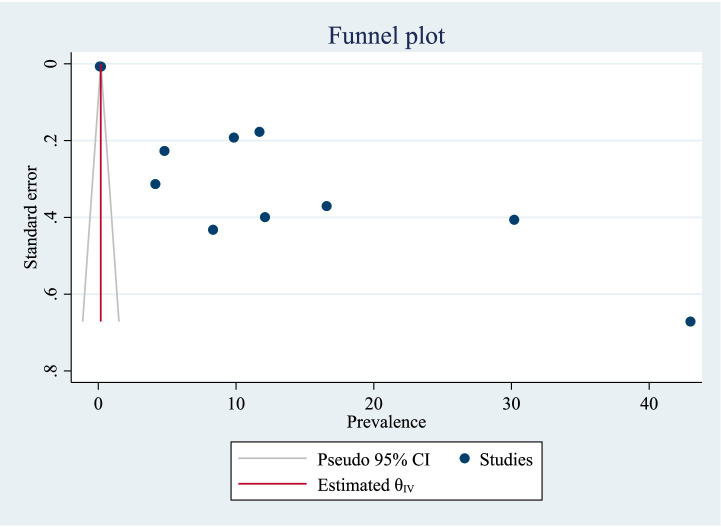
Funnel plot for Publication bias of studies for prevalence of TB.

A funnel plot was utilized for analyzing potential publication bias in the identified study. As a consequence, the visually viewed funnel plot exhibited asymmetry, indicating that publication bias existed among researches. The listed papers’ possible publication bias was assessed using Egger’s test. With a *p*-value of 0.00, the Egger’s test of TB prevalence revealed publication bias. Additionally, the visually assessed TB prevalence funnel plot revealed publication bias (Figure 2). A trim and fill analysis was carried out to estimate the number of potentially missing studies in order to minimize and account for the observed publication bias in the studies. Trim and fill analysis yielded an estimated pooled prevalence of TB of 15.626 (95% CI = 8.129–23.122) after controlling for publication bias (Table 2).

**Table 2:** Trim-and-fill analysis for the prevalence of TB.

Studies	Prevalence	[95% CI]
Observed	12.810	5.070–20.549
Observed + Imputed (11 + 2)	15.626	8.129–23.122

##### Sensitivity

**Figure 3:**
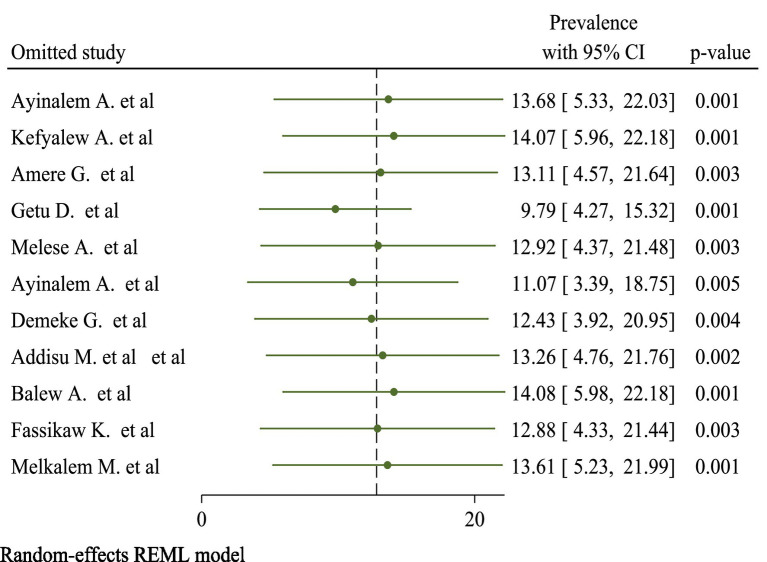
Sensitivity analysis result of included studies on prevalence of TB.

In sensitivity analyses using the leave-one-out strategy, eliminating none of the studies had a significant influence on pooled burden estimates and heterogeneity measures within review studies. As a result, sensitivity analysis using the random-effects model revealed that no single study altered the overall prevalence of TB (Figure 3).

#### Pooled prevalence of TB in Ethiopia

**Figure 4:**
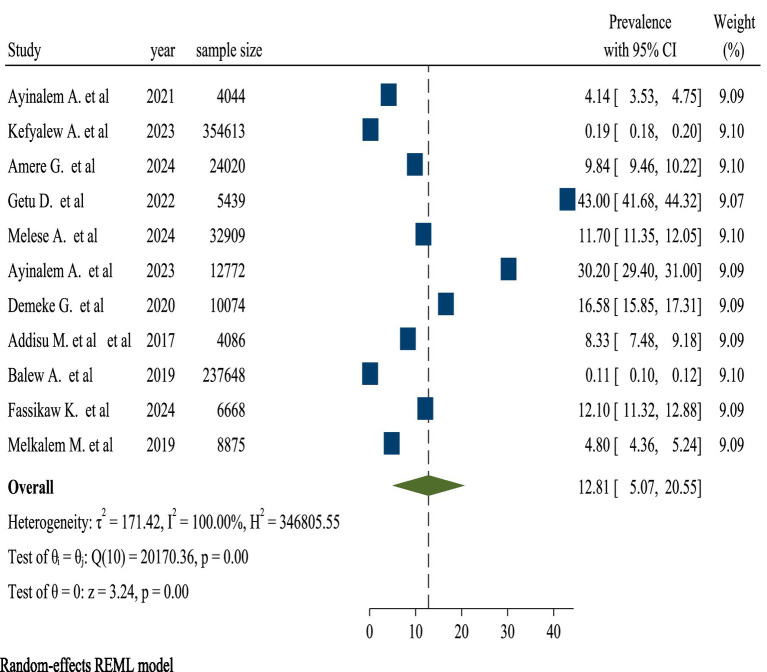
Forest plot for the pooled prevalence of TB.

The pooled TB prevalence was 12.81% (95% confidence interval, 5.07–20.55). A random-effects model reveals heterogeneity among studies on the prevalence of TB with a 95% confidence interval (I^2^ = 100% and *p*-value = 0.00). Because of the high heterogeneity between the included studies, subgroup analysis was carried out to know the prevalence of TB among review articles (Figure 4).

#### Subgroup analyses of TB prevalence in Ethiopia by sample size and number of studies

**Figure 5:**
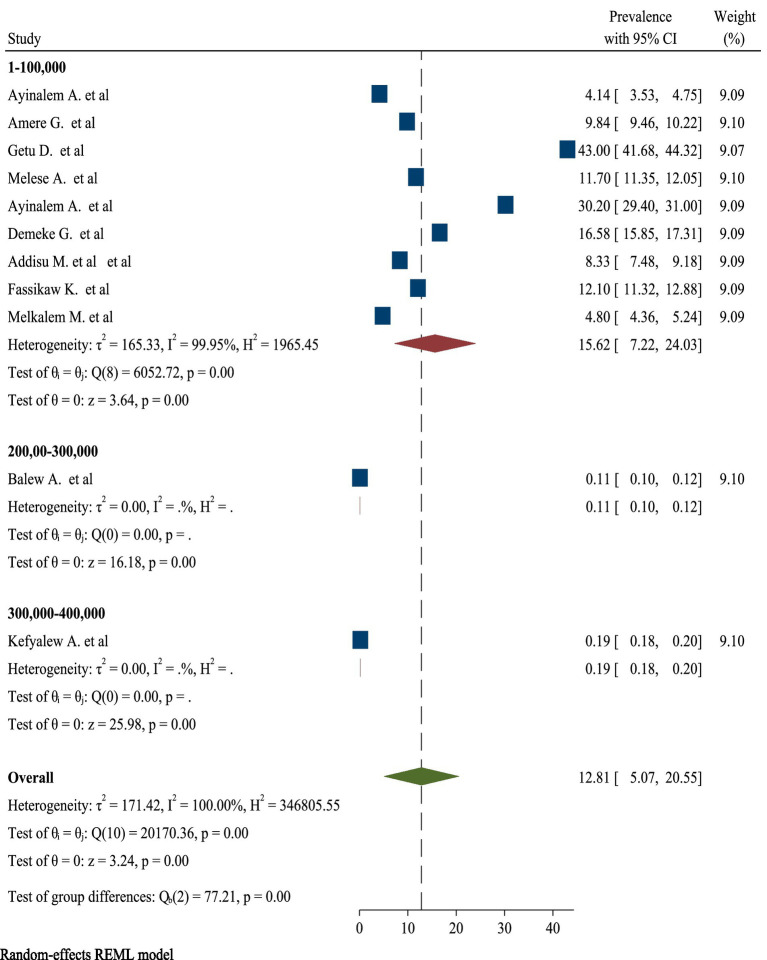
Subgroup analyses for pooled prevalence of TB based on number of sample size.

**Figure 6:**
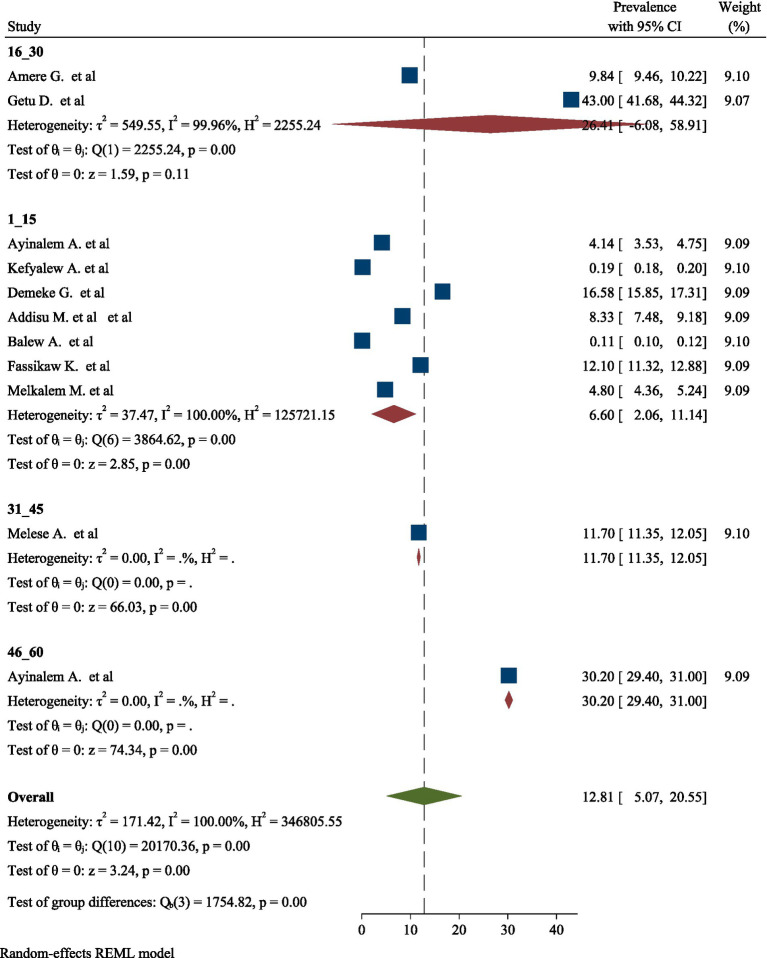
Subgroup analysis for pooled prevalence of TB based on number of studies.

High significant heterogeneity was found among included studies regarding TB prevalence. Inverse of variance (I^2^) statistics revealed greater than or equal to 100% heterogeneity among studies for TB prevalence. Subgroup analysis was conducted for TB prevalence on number of sample size and number of studies in order to determine the potential source of heterogeneity. As a result, the analysis revealed a significant difference in TB frequency among studies on number of sample size and number of studies (Figures 5, 6), respectively.

## Discussion

Tuberculosis is the most common cause of mortality and morbidity among transmissible illnesses across the world. According to the WHO’s 2018 global TB report, 10 million individuals acquired TB and 1.6 million died in 2017, including 300,000 fatalities among HIV positive patients (28). Ethiopia is one of the least developed nations in the world and one that has been severely affected by TB outbreaks. The country recorded 117,705 TB cases, 28,600 deaths, and just 11% of the $93 million required annually for TB care and control came from domestic sources, according to the 2018 global TB report (28). As a result, national prevalence surveys are critical for providing a more accurate estimate of a country’s TB burden and demonstrating the success or failure of TB prevention and control efforts.

In this umbrella review, the weighted pooled prevalence of TB in Ethiopia was 12.81% (95% CI, 5.07–20.55). This finding was inline with that reported in Ethiopia (8.9, 13.3, 7.8, 10.5, 9.7%) (29–33). This might be due to socioeconomic conditions, such as poverty and overcrowded living environments, limited access to healthcare services, including inadequate infrastructure and a lack of awareness about TB symptoms. Similarly, this finding was inline with that reported in China (6.0%) (34), in the world (15.0%) (35), endemic settings (13.5%) (36), and sub-Saharan Africa (7.74 and 20%) (37, 38). The comparability of data on TB prevalence in these studies can be attributed to several key reasons. All sorts of research frequently conform to stringent methodological requirements, ensuring that data gathered from multiple sources is look over properly. This involves choosing research based on predetermined criteria, which helps to ensure consistency in the sorts of people and settings included in the analysis. The use of uniform definitions and diagnostic criteria for TB across research improves comparability, allowing for more precise aggregate of prevalence rates. Furthermore, these studies usually analyze the quality of the included research, which helps to reduce biases and variations in data reporting. Finally, focusing on certain geographical locations or demographic groups might yield similar results since these factors frequently impact the frequency of TB in comparable ways. Overall, these characteristics help to provide a more consistent picture of TB prevalence across study (39).

Conversely, this finding was lower than that reported in Ethiopia (25.4, 30.5%) (40, 41). This could be attributed to enhanced public health initiatives, greater awareness and education regarding TB prevention and symptoms, as well as advancements in healthcare infrastructure and improved access to medications, may be highlighted in this review of various studies. Likewise, this finding was lower than that reported in China Africa (30%) (42), India (36, and 41%) (43, 44), low and middle income countries (79.6%) (45), and in the World (32.6%%) (46). The high and heterogeneous distribution of TB incidence rates across these countries and African subdivisions could be attributed to inconsistency in problem-solving due to poorly tracked and incomprehensible strategies, a lack of collaboration between donor countries and multilateral institutions to address TB and HIV infection, famine, conflict, and drought. This necessitates the cooperation of foreign donor agencies such as WHO and United States Agency for International Development in a coordinated strategy that encompasses both infection testing and treatment. Prospective cohort studies revealed a greater prevalence of TB among HIV patients (47–49).

However, this finding was higher than that reported in Ethiopia (1.8, 1.6, 1.1%) (50–52). This may be attributed to the resurgence of TB, driven by various socio-economic challenges such as poverty and overcrowding. Furthermore, limited access to healthcare and inadequate public health initiatives can exacerbate the spread of the disease, may be suggested by the studies included in this review. Also, this finding was higher than that reported in sub-Saharan Africa (4.02%) (53) and in African and Asian countries (4.72%) (54). These discrepancies might be due to socioeconomic disparities between developed and developing nations, the high prevalence of TB in the study region, and the progressive transformation of latent TB into active TB disease, which leads to an epidemic of TB in the study region relative to higher-income nations. They could also be explained by differences in the execution and policies of the health system (55, 56).

This review found high heterogeneity in the prevalence of TB, with I^2^ statistics showing values more than or equal to 100% at *p* = 0.00. To account for this heterogeneity, a subgroup analysis was conducted based on number of sample size and number of studies. Despite subgroup analysis, there was still considerable heterogeneity. This continued inconsistency might be attributable to a variety of variables, including changes in research design, population demography, and TB diagnostic and reporting methodologies. Furthermore, regional variables, such as urban vs. rural settings, as well as socioeconomic considerations, may contribute to observed disparities in prevalence rates. Furthermore, the time period of data collection and the incidence of co-morbidities in study populations might further confound the comparability of results, underscoring the complexity of TB distribution across various contexts (57, 58).

Moreover, this umbrella review demonstrated a significant difference in the prevalence of TB among studies when analyzed by sample size. The findings indicated that studies with larger sample sizes tended to report different prevalence rates compared to those with smaller samples. This discrepancy may arise from several factors, including the increased statistical power and representativeness of larger studies, which can better capture the true burden of TB in diverse populations. Conversely, smaller studies may be more susceptible to sampling bias and may not adequately reflect the broader epidemiological trends. Additionally, variations in the methodologies employed, such as differences in diagnostic criteria and data collection techniques, could further contribute to the observed differences in prevalence. Overall, the analysis underscores the importance of considering sample size as a critical factor influencing the reported prevalence of TB in research studies (59, 60).

Likewise, this umbrella review revealed a significant difference in the prevalence of TB among studies based on the number of studies included in the analysis. The findings indicated that studies with a higher number of contributing research articles tended to show variations in prevalence rates compared to those with fewer studies. This discrepancy may be attributed to the diverse methodologies, populations, and settings represented in the larger pool of studies, which can lead to a broader range of reported prevalence rates. Furthermore, the inclusion of studies from different geographical regions and varying health care systems can introduce additional variability in the results. The differences observed highlight the importance of considering the quantity and quality of studies when interpreting prevalence data, as a more extensive body of research can provide a more comprehensive understanding of TB epidemiology. Overall, this analysis emphasizes the need for careful consideration of study selection in systematic reviews and meta-analyses to ensure accurate representation of TB prevalence (61, 62).

As far as we are aware, this is the first comprehensive overview of the prevalence of TB in Ethiopia. We used a predetermined data abstraction methodology and search technique. We used widely accepted techniques for assessing the quality of individual studies as well as critically analyzing these studies.

One significant limitation of this umbrella review is the small number of articles included in the analysis, which may have an impact on the findings’ robustness and generalizability. The limited number of systematic reviews and meta-analyses on TB prevalence in Ethiopia limits the capacity to make generalizations about the disease burden in various demographics and settings. As a result, this shows a crucial gap in the existing study, underscoring the urgent need for more high-quality, large-scale studies that can provide stronger evidence. Future study should focus on undertaking well-designed epidemiological studies to enhance our understanding of TB prevalence and determinants in Ethiopia. By addressing this limitation, future umbrella reviews may provide more conclusive insights and encourage public health strategies focused on controlling and eliminating TB in the region.

## Conclusions and recommendations

The pooled prevalence of TB in Ethiopia is higher, despite the limited data available to establish and generate strong evidence. As a result, national TB control programs should pay due attention and implement appropriate control measures, such as regular systematic TB screening and mandatory TB testing for probable TB cases. Moreover, coordination at the local, national, and international levels is essential to address the variables that cause TB and reduce its impact on public health. It also provides valuable information to Ethiopian officials and policymakers.

## Data Availability

The original contributions presented in the study are included in the article/supplementary material, further inquiries can be directed to the corresponding author.
